# Validation of Motion Tracking Software for Evaluation of Surgical Performance in Laparoscopic Cholecystectomy

**DOI:** 10.1007/s10916-020-1525-9

**Published:** 2020-01-24

**Authors:** Sandeep Ganni, Sanne M. B. I. Botden, Magdalena Chmarra, Meng Li, Richard H. M. Goossens, Jack J. Jakimowicz

**Affiliations:** 1 Delft University of Technology, Industrial Design Engineering, Medisign, Delft, The Netherlands; 2 GSL Medical College, Department of Surgery, Rajahmundry, India; 3 Catharina Hospital, Research and Education, Michelangelolaan 2, 5653 EJ Eindhoven, The Netherlands; 4 Department of Pediatric Surgery, Radboudumc – Amalia Children’s Hospital, Nijmegen, the Netherlands

**Keywords:** Motion tracking, Objective evaluation, Indices of performance, Laparoscopic skills training, Video-based assessment, Thresholds of performance

## Abstract

Motion tracking software for assessing laparoscopic surgical proficiency has been proven to be effective in differentiating between expert and novice performances. However, with several indices that can be generated from the software, there is no set threshold that can be used to benchmark performances. The aim of this study was to identify the best possible algorithm that can be used to benchmark expert, intermediate and novice performances for objective evaluation of psychomotor skills. 12 video recordings of various surgeons were collected in a blinded fashion. Data from our previous study of 6 experts and 23 novices was also included in the analysis to determine thresholds for performance. Video recording were analyzed both by the Kinovea 0.8.15 software and a blinded expert observer using the CAT form. Multiple algorithms were tested to accurately identify expert and novice performances. ½ L + $$ \raisebox{1ex}{$1$}\!\left/ \!\raisebox{-1ex}{$3$}\right. $$ A + $$ \raisebox{1ex}{$1$}\!\left/ \!\raisebox{-1ex}{$6$}\right. $$ J scoring of path length, average movement and jerk index respectively resulted in identifying 23/24 performances. Comparing the algorithm to CAT assessment yielded in a linear regression coefficient R^2^ of 0.844. The value of motion tracking software in providing objective clinical evaluation and retrospective analysis is evident. Given the prospective use of this tool the algorithm developed in this study proves to be effective in benchmarking performances for psychomotor skills evaluation.

## Introduction

Training and assessment in laparoscopic surgery are increasingly moving towards more objective and criterion-based evaluation tools. [1–3] Box trainers with cameras, virtual and augmented reality simulators have facilitated in achieving objective evaluation of technical skills. [4–7] Recent trends in surgical training, such as self-directed learning and reflective practice, indicate a positive effect of repetitive and independent practice, which have been made possible with objective evaluation tools. [8–10] Several objective criteria such as instrument movement, procedure time, and procedure specific risky maneuvers can be extracted from these simulators and serve as benchmarks for assessing the performance or self-assessment for progress monitoring. [11, 12] However, the use of these objective criteria in the operating room to assess real surgical procedures is currently limited.

It has been proven by Yamaguchi et al. that motion tracking of the surgical instruments can objectively differentiate between expert and novice surgeons in a skills lab setting. This has been achieved using specialized instruments using motion trackers and cameras. [13–16] We have previously used a motion tracking software which is independent of specialized equipment and instruments during the procedure and can be used for retrospective performance analysis using the video recording of the procedure. [17] In this previous study three indices were identified, namely ‘path length’, ‘sudden movements’ and ‘average movements’, which could be extracted from the recorded videos classify expert and novice performances. These indices, however, were procedure specific and as such required a set of benchmarks to assess individual procedures.

Recent advances in image recognition and artificial intelligence (AI) have been proven effective in surgical skills evaluation. [18, 19] These systems are more task and procedure specific, because they evaluate the surgical skills required for laparoscopic knot tying, suturing or pelvic lymph node dissection. But, as with any laparoscopic surgery, skills are broadly categorized into cognitive and psychomotor skills. Cognitive skills as such are procedure specific and psychomotor skills are pan-procedural. Thus, the aim of this study is to develop a new set of benchmarks for psychomotor skills that scale between novice and expert performance and can be used in automated assessment tools.

## Methods

### Protocol

To determine a good threshold for the algorithm, the data has to be categorized as shown in Table 1. To determine these thresholds, the data from our previous study [17] was evaluated and recalculated. Three parameters were calculated: ‘Path length’ (L); ‘Average distance’ (A), which the instrument tip moved per time frame; and ‘Number of extreme movements’ (J), defined as more than 1.0 cm movement per frame. If the value of the parameter was above the expert median, a score of 1 was assigned, if it was below the novice median, a score of 0 was assigned. Scores between the two medians were assigned a score between 0 and 1, scaled linearly. Following, these scores were weighted using the following equation, to create a total performance score (p), ranging from 0 to 1:

**Table 1:** Ideal thresholding output from the algorithm

Threshold	Category	Procedures performed
p > =2/3	Expert	200 or more procedures
p < =1/3	Novice	10 or fewer procedures

*w*_*l*_, *w*and*w*_*j*_, where *w*_*l*_ + *w*_*a*_ + *w*_*j*_=^1^thus:

1 $$ {w}_lL+{w}_aA+{w}_jJ:= \rho $$

The aim of this study was to calculate the best weightings to determine expertise in uncomplicated laparoscopic cholecystectomy procedure.

First the original participant data from our previous study was used to determine the expertise thresholds as described above. [17] Following, a blinded evaluation of twelve new videos was performed by both the tracking system and the Competency Assessment Tool (CAT) for laparoscopic cholecystectomy by a blinded assessor to correlate the data. The videos were rated with the new weighting equation and evaluation for a significant correlation. These results were then compared to the previously recorded experience of the surgeon or surgical resident performing the procedure to determine whether the algorithm had correctly identified their level of psychomotor skills expertise.

### Participants

This study uses data from the six ‘experts’ (>200 laparoscopic procedures performed) and 23 ‘novices’ (<10 laparoscopic procedures performed but with a surgical background) in our previous study, to create thresholds for expertise. [17] These thresholds were then tested on an additional twelve blinded video recordings of six surgeons and six surgical residents, conducting an uncomplicated laparoscopic cholecystectomy procedure at the Catharina Hospital, Eindhoven, The Netherlands. This was to assess, by blinded trial, the ability of this thresholding algorithm in determining the psychomotor skills demonstrated in the procedure. All participants gave their consent for the video recording of the procedures used in this study and hospital ethics committee approval was obtained.

### Data extraction and statistics

The tracking data of the instrument movements during the surgical procedure was extracted from the recorded videos using Kinovea 0.8.15 software. Both the thresholding calculations and extracted data were analyzed, including linear regression analysis, using MATLAB (R16b).

## Results

### Threshold Determined

Data from the tracking software was processed using the thresholding function and Equation described in the methods section, various weightings were evaluated and compared to the correct categorization to identify the best assessment algorithm (Table 2).

**Table 2:** The values of the weighting parameters for the thresholding and the corresponding number of correctly identified experts and novices

Set	Path length (L)	Average distance (A)	Extreme movements (J)	Correctly Identified
1	1/3	1/3	1/3	20/24
2	1/3	1/6	1/2	18/24
3	1/3	1/2	1/6	19/24
4	1/6	1/3	1/2	15/24
5	1/2	1/3	1/6	23/24
6	1/6	1/2	1/3	18/24
7	1/2	1/6	1/3	21/24

Set 5 resulted in the most correctly categorized videos, which concluded in the following Algorithm:

Assessment score (0–1): Score = ½ L + $$ \raisebox{1ex}{$1$}\!\left/ \!\raisebox{-1ex}{$3$}\right. $$A + $$ \raisebox{1ex}{$1$}\!\left/ \!\raisebox{-1ex}{$6$}\right. $$ J

### Validity of assessment algorithm

Twelve videos were analyzed using the new algorithm with the tracking system and scored using the CAT form by a blinded expert assessor. The thresholding algorithm categorized the twelve videos as five experts, five intermediates and two novices. The expert-assigned CAT scores support this ordering as shown in Table 3. Upon unblinding the data, all the videos identified as expert videos were indeed performed by experienced surgeons and had the top four CAT scores. The other videos evaluated were in fact performances of surgical residents with an intermediate or novice level. Those identified as novices by the algorithm scored the lowest CAT score assigned by the expert assessor. One surgeon was identified as intermediate according to the algorithm, but also scored the lowest CAT score of the surgeons and had a very high jerk index.

**Table 3:** The weighted score is the score calculated using the data extracted for the video and the thresholding equation, performance algorithm

Video	Score performance algorithm	Category Identified by thresholds	CAT Score	Actual video category
1	1.00	Expert	21	Surgeon
2	1.00	Expert	22	Surgeon
3	1.00	Expert	20	Surgeon
4	0.86	Expert	19	Surgeon
5	0.67	Expert	20	Surgeon
6	0.63	Intermediate	19	Surgeon
7	0.54	Intermediate	17	Resident
8	0.41	Intermediate	14	Resident
9	0.36	Intermediate	14	Resident
10	0.35	Intermediate	13	Resident
11	0.09	Novice	14	Resident
12	0.00	Novice	13	Resident

Along with the category that this score yields (from Table 1). The Expert CAT score for that video is also shown and whether the video was, in fact, performed by an experienced surgeon or a student

### Significance level

**Fig. 1:**
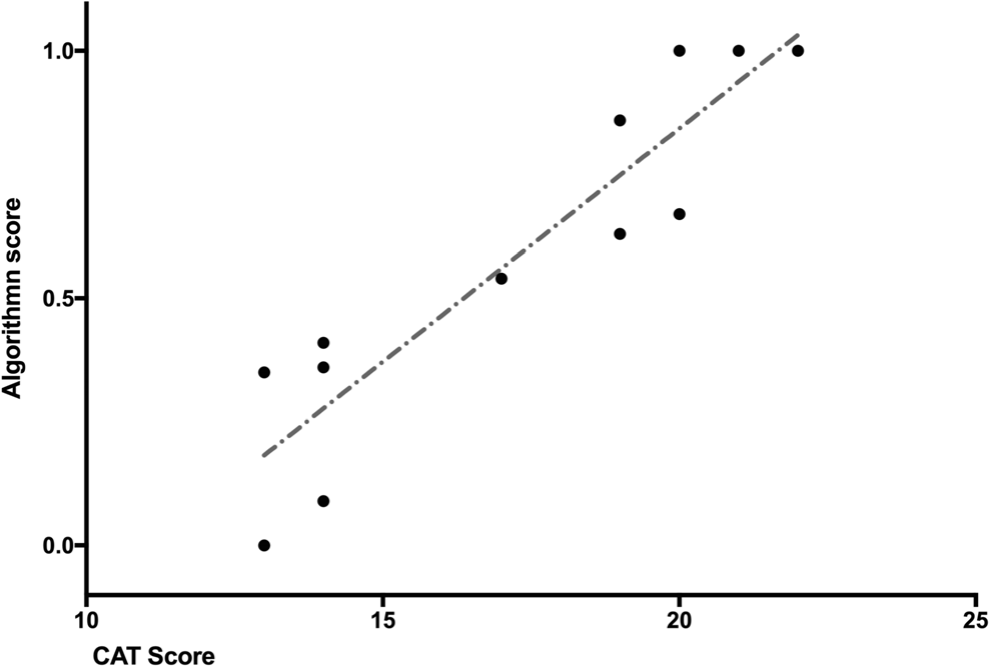
Plot of Weighted score of videos, p vs expert-assessed CAT score. The linear trendline has a regression coefficient of determination (R^2^) of 0.844

The CAT Tool is a comprehensive assessment tool that assesses performance across the three tasks in laparoscopic cholecystectomy in exposure of the cystic duct and artery, cystic pedicle dissection and resection of the gallbladder. [20] These tasks are further evaluated across different indices such as usage of instruments, handling of tissue, errors occurred and the end-product. For this study, we only considered the scoring across the usage of instruments and handling of tissue as they determine the psychomotor skills. Figure 1 depicts the linear regression curve plotted using the CAT score and the algorithm yielding a coefficient R^2^ of 0.844.

### Performance scoring

Scoring systems provide reference for ideal performance and serve as an indicator for measuring learning curve progression and consistency in performance. Upon analysis of the results from the algorithm and correlation with the CAT we propose the following range of scores as derived when using the algorithm for assessing psychomotor skills in laparoscopic cholecystectomy:

*Expert performance:* 0.65 and above

*Intermediate performance:* 0.35–0.65

*Novice performance:* 0.35 and below

## Discussion

Traditionally assessing surgical skills requires expert assessment through standardized validated tools such as the Competency Assessment Tool (CAT) and Objective Structured Assessment of Technical Skills (OSATS) [20–22]. Objective evaluation of laparoscopic skills using motion analysis has been limited to VR simulators and robotic surgery [23]. The transfer of these evaluation criteria to clinical laparoscopic surgery has been limited by the use of additional equipment and costs [24].

Computer vision techniques and AI have shown promising results in identifying procedure specific evaluations [18, 19]. Their strengths lie in detecting cognitive and clinical skills in addition to error recognition. AI can also effectively segment procedural steps for easy access and indexing for future reference [25]. However, these systems do not identify psychomotor skills that can be applied pan procedurally which can serve as an important indicator for learning curve monitoring in the clinical context.

Based on our previous study on the feasibility of the Kinovea software [17], the thresholds for the expertise levels were determined using results therefrom. This study was procedure-specific using uncomplicated laparoscopic cholecystectomy in the clinical setting. The thresholds were set based on a new algorithm, which was validated by comparing it with both objective expert assessors (*p* = 0.01, R^2 = 0.844). Overall, the current threshold algorithm seems to provide a potential objective assessment tool for psychomotor skills evaluation. The algorithm is weighted on the importance of each of the indices identified and the rate in which these make up the expertise of the performance.

However, this study has shown the potential value of the Kinovea tracking software to rapidly evaluate one’s psychomotor skills automatically of a laparoscopic procedure, retrospectively, without the need for additional equipment during the procedure. Moreover, because the scoring is by assessing surgical videos retrospectively, there is no need for the use of other equipment or the stress of being watched by an assessor. Surgical trainees in a skills lab setting are used to objective metric scores as part of their self-improvement on VR and AR simulators and this new assessment method could be developed to act as a bridge to clinical settings; having value in both self-assessments, for improving the learning curve and as a tool for measuring psychomotor skills.

### Limitations

Whilst the algorithm presents a promising first step towards bridging the gap between true objective evaluation from the skills lab to the operating theatre, the current calculations used in this study are limited in their application to assessing psychomotor skills required for laparoscopic cholecystectomy. Furthermore, as they represent a broad average of movement, these indices do not currently provide an indication of errors or potential errors. However, in combination with computer vision techniques and AI that are proven to recognize procedure and task specific errors based on image recognition, this algorithm could in the future be developed to serve in providing a more comprehensive evaluation of laparoscopic skills, similar to that of VR simulators, in a clinical setting. Furthermore, with the new insights of this study in the categorization of the importance of performance indices, it could be transferred to other laparoscopic procedures.

## Conclusion

The value of motion tracking software in providing objective clinical evaluation and retrospective analysis is evident. Given the prospective use of this tool the algorithm developed in this study proves to be effective in benchmarking performances for psychomotor evaluation of laparoscopic skills.
